# A two-tier protection strategy for oral delivery of GLP-1 peptides: lipid-based formulation combined with enteric capsules

**DOI:** 10.3389/fddev.2026.1770094

**Published:** 2026-05-22

**Authors:** Camille Dumont, Vanessa Gonzalez, Paula Klatt, Sandrine Picco, Delphine Nombret, Marine Agisson, Euengla Uku, Thibault Saurel, Vincent Jannin

**Affiliations:** Capsugel France SAS, Colmar, France

**Keywords:** customized capsule, enteric delivery, lipid-based formulations, oral peptide delivery, permeation enhancer

## Abstract

**Introduction:**

Oral delivery of peptides is a promising alternative to invasive injectable therapies, offering improved patient adherence and convenience. Glucagon-like peptide-1 (GLP-1) analogs, in particular, are highly effective for the management of type 2 diabetes mellitus and obesity, yet their oral bioavailability remains limited by enzymatic degradation and poor intestinal permeability.

**Methods:**

In this study, we aimed to design a lipid-based formulation (LBF) of the model GLP-1 analog exenatide (EXE) in its hydrophobic ion-paired (HIP) form, incorporating permeation-enhancing excipients, and evaluate its performance in enteric capsule systems.

**Results and discussion:**

EXE-HIP was prepared with sodium docusate (1:4 molar ratio), which increased lipophilicity (log P = 0.9 ± 0.2 vs. −2.9 ± 0.3 for EXE) and markedly improved solubility in lipid excipients, especially in Labrafac™ MC60, a known promoter of intestinal permeability. An optimized LBF composed of 85% Labrafac™ MC60, 10% Kolliphor® RH40, and 5% propylene glycol was developed, with a final particle size of 106 nm, and it was loaded with 6 mg/g solubilized EXE (HIP form) and 20 mg/g of sodium caprate (complementary permeation enhancer). The formulation exhibited significant protection against α-chymotrypsin-mediated degradation, which is consistent with sequestration of EXE-HIP within lipid droplets that limit enzymatic access. Short-term stability studies highlighted formulation-dependent degradation behavior, with higher impurity formation observed for solubilized EXE-HIP than for suspended EXE acetate, particularly at room temperature. To further support oral delivery, customized gelatin/HPMC-AS enteric capsules were engineered to provide a complementary two-tier protection strategy: (i) protecting the peptide and lipid phase from gastric enzymatic degradation and preventing premature dilution in gastric fluids that could displace the peptide from its protective lipid environment, while (ii) ensuring coordinated release of both EXE-HIP and the permeation enhancers at the intestinal absorption site. These capsules met the USP delayed release requirements. Overall, this work presents a promising proof-of-concept oral delivery platform that combines HIP chemistry, lipid-based formulation, and ready-to-use enteric capsule technology to enhance *in vitro* enzymatic protection of peptide therapeutics, thereby laying the groundwork for future studies to assess bioavailability and therapeutic efficacy *in vivo*.

## 1 Introduction

Oral delivery of peptides has been a major research focus for several decades, driven by the promise of replacing injectable therapies with more convenient and patient-friendly dosage forms. This long-standing effort culminated in the late 2010s with the approval of Rybelsus® (semaglutide, Novo Nordisk) and Mycapssa® (octreotide, Chiasma), which demonstrated that effective oral peptide delivery is achievable at the commercial scale. Beyond improving convenience and reducing the treatment burden, oral peptide products can also broaden access to therapy for individuals who experience needle aversion and may otherwise delay or avoid care (Alsbrooks and Hoerauf, 2022).

Among the therapeutic areas where oral delivery could have a transformative impact, glucagon-like peptide-1 (GLP-1) analogs stand out. These peptides provide highly effective treatment options for type 2 diabetes mellitus and obesity, but their current reliance on subcutaneous injection can adversely affect adherence, particularly during long-term therapy. Transitioning GLP-1 treatments to the oral route would, therefore, represent a major advancement. However, achieving efficient oral peptide delivery remains challenging as peptides are rapidly degraded by gastrointestinal enzymes, their hydrophilic nature limits transcellular permeation, and their relatively large molecular size restricts paracellular transport. These barriers highlight the need for innovative formulation and delivery strategies that are capable of ensuring reliable intestinal uptake.

Lipid-based formulations (LBFs) offer a promising approach for overcoming these obstacles. By maintaining peptides solubilized within the lipid phase, LBFs can protect them from proteolytic degradation in the gastrointestinal tract, provided that peptide lipophilicity is sufficiently increased (Hetényi et al., 2017). Hydrophobic ion pairing (HIP) offers an efficient and chemistry-preserving method for achieving this transformation (Griesser et al., 2017; Ristroph and Prud’homme, 2019). LBFs can also enhance intestinal permeability, particularly when formulated with medium-chain fatty acids, either as specific permeation enhancers such as sodium caprate (Maher et al., 2009; Twarog et al., 2022; 2021; 2019) or as lipid excipients containing these fatty acids (McCartney et al., 2025; 2024; 2019). Moreover, neutral or negatively charged LBFs with particle sizes below 100 nm can improve transport through the mucus layer (Griesser et al., 2018), thereby promoting close interaction with the intestinal epithelium.

The combination of peptide HIP and LBF has shown substantial improvements in oral bioavailability in preclinical models (Bonengel et al., 2018; Li et al., 2021; Menzel et al., 2018; Venkatasubramanian et al., 2025); however, these studies typically relied on oral gavage, which exposes formulations to large volumes of gastric fluid. Such dilution increases the risk of premature peptide partitioning from the lipid droplets and subsequent enzymatic degradation (Chamieh et al., 2019). Encapsulating LBFs in enteric capsules offers a way to mitigate these challenges. The enteric capsule shell provides a physical barrier against gastric peptidases and prevents dilution-driven destabilization of the formulation, enabling intact delivery to the small intestine where lower fluid volumes favor close mucosal contact. This administration mode also reduces the risk of separating the active pharmaceutical ingredient (API) from the permeation enhancer in gastric fluids and ensures their localized co-release at the absorptive site, thereby supporting efficient uptake.

To enable such targeted delivery, Capsugel has developed the Capsugel® Enprotect® capsule, a ready-to-fill enteric capsule designed to preserve the stability and biological activity of sensitive macromolecules in biorelevant media (Jannin et al., 2023). This capsule has demonstrated robust gastro-resistance under both fasted and fed conditions in healthy volunteers (Grimm et al., 2023; Rump et al., 2022). Its patented bilayer structure, composed of a hydroxypropyl methylcellulose (HPMC) structural layer and a hydroxypropyl methylcellulose acetate succinate (HPMC-AS) functional layer, enables precise release of the payload in the ileum. This versatile technology allows fine-tuning of the target release profile and compatibility with the formulation by adjusting the composition of the capsule shell.

The objectives of this study were twofold. First, we aimed to develop a state-of-the-art lipid-based formulation of a model GLP-1 analog, exenatide (EXE), which is formulated as a HIP and combined with a permeation enhancer to achieve a low-particle-size dispersion optimized for intestinal absorption. Second, we sought to evaluate the enteric performance of the Capsugel® Enprotect® capsule when filled with this optimized LBF and, if necessary, develop a customized enteric capsule compatible with the formulation to ensure robust gastro-protection and targeted small-intestinal delivery.

## 2 Materials and methods

### 2.1 Materials

EXE acetate (Batch AS521560) was purchased from Apollo Scientific (Manchester, United Kingdom). Sodium docusate (#BCCD8614), sodium lauryl sulfate salt (#SLCM6410 and SLCL9622), sodium caprate (#SLCR4016), butylated hydroxyanisole (BHA, #SHBR4871), butylated hydroxytoluene (BHT, #STBL0486), propyl gallate (#MKCV7754), and α-chymotrypsin from bovine pancreas type II (#0000349760) were purchased from Sigma-Aldrich (Saint Louis, MO, United States). Ascorbyl palmitate (#LRAD9010) from Merck (Darmstadt, Germany) and DL-α-tocopherol (N10I032) was purchased from Thermo Fisher Scientific (Oxford, United Kingdom). Esomeprazole magnesium trihydrate (EMT)-coated sucrose beads (#VL5-005) were purchased from Xedev (Zele, Belgium). Size #0 Capsugel® Enprotect® capsules (#54267561) were manufactured by Capsugel France SAS (Colmar, France). Information of the lipid excipients is provided in Table 1.

**TABLE 1:** References of lipid excipients used in this study.

Commercial name	Chemical name (USP NF)	Batch number	Supplier	Peroxide value^a^ (meqO_2_/kg)
Capryol® 90	Propylene glycol monocaprylate	200455	Gattefossé^b^	0.4
Glycerin	Glycerin	22,020,054	Cooper^c^	NA
Kolliphor® ELP	Polyoxyl 35 castor oil	16012447G0	BASF^d^	NA
Kolliphor® PS20	Polysorbate 20	0028310598	BASF	<1.0
Kolliphor® PS80	Polysorbate 80	0027859005	BASF	<1.0
Kolliphor® RH40	Polyoxyl 40 castor oil	29226716K0	BASF	NA
Kollisolv® PEG E 400	Polyethylene glycol 400	96036424U0	BASF	NA
Labrafac™ MC60	Glyceryl monocaprylocaprate	192936 and 199348	Gattefossé	<0.1 and <0.1
Labrafil® M1944CS	Oleoyl polyoxyl-6 glycerides	187704	Gattefossé	<0.1
Labrasol® ALF	Caprylocaproyl Polyoxyl-8 glycerides	193114 and 200713	Gattefossé	<0.1 and <0.1
Lauroglycol® 90	Propylene glycol monolaurate	186707 and 201953	Gattefossé	<0.1 and 0.4
Maisine® CC	Glyceryl monolinoleate	185033 and 199163	Gattefossé	0.3 and <0.1
Propylene glycol	Propylene glycol	21100045	Cooper	NA
Soybean oil	Soybean oil	0001329404	Olvea^e^	0.1

NA, not available.

^a^ As reported on the Certificate of Analysis.

^b^ Gattefossé SAS (Saint Priest, France).

^c^ Cooper (Melun, France).

^d^ BASF (Ludwigshafen, Germany).

^e^ Olvea (Saint Leonard, France).

### 2.2 Methods

#### 2.2.1 Exenatide quantification and assay methods

##### 2.2.1.1 Quantification method

EXE was quantified by high-performance liquid chromatography (HPLC) using an Alliance system from Waters (Dachstein, France) equipped with a UV-DAD detector (W2998, Waters) set at 280 nm. Separation was carried out on an XBridge C18 3.5 μm 4.6 × 150 mm column (Waters) at 40 °C. A linear gradient of elution was applied from 60% solvent A/40% solvent B to 34% solvent A/66% solvent B [solvent A: 0.1% (v/v) TFA in water/solvent B: 0.1% TFA in water (20%): acetonitrile (80%)]. The flow rate was 0.6 mL/min, and the injection volume was 20 µL. The retention time was approximately 8.9 min. The response of EXE in the diluent was linear from 2.969 μg/mL to 989.802 μg/mL. Therefore, the lower limit of quantification (LOQ) was set at 3 μg/mL.

##### 2.2.1.2 Assay RS method

The EXE assay method was adapted from the EXE USP monograph. EXE and relative substances (RSs) were quantified by HPLC equipped with a UV-DAD detector (W2998) set at 220 nm. Separation was carried out on an XBridge C18 3.5 μm 4.6 × 150 mm column (Waters) at 60 °C using the elution gradient described in Table 2 between solvent A (10 mM ammonium hydrogen carbonate, pH 9.5, 0.0375% ammonia) and solution B (90% Acetonitrile/10% solution A). The flow rate was 0.8 mL/min, and the injection volume was 10 µL. EXE was extracted from the lipid matrix by diluting 200 mL samples in 5 mL methanol prior to HPLC quantification.

**TABLE 2:** Elution gradient of the assay RS method.

Time (min)	Solution A (%)	Solution B (%)
0	74	26
0.5	74	26
33	63	37
35	10	90
36	10	90
36.1	74	26
42	74	26

#### 2.2.2 HIP formation/precipitation efficiency

**FIGURE 1:**
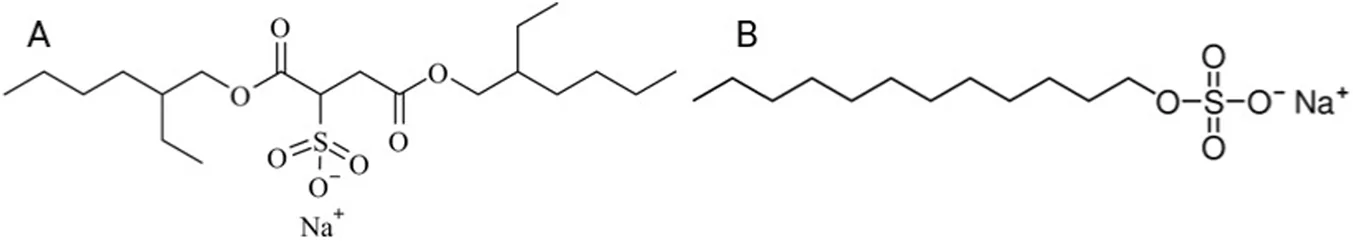
Chemical structures of **(A)** sodium docusate and **(B)** sodium lauryl sulfate.

The ability of sodium docusate (DOC) and sodium lauryl sulfate (SLS) to form hydrophobic ion pairs (HIPs), also called lipophilic salts, with EXE was evaluated in the EXE:counter-ion molar ratios of 1:1, 1:2, 1:4, 1:6, and 1:8. Chemical structures of DOC and SLS are reported in Figure 1.

Stock solutions of 10 mg/mL EXE in Milli-Q water were prepared and acidified by the addition of HCl 1 N solution to reach a pH value below 2.8. Ionization of the peptide was confirmed by the observation of transparent solutions. Aqueous solutions of DOC and SLS were prepared so that the desired molar ratios were obtained for iso-volumes addition of counter-ion and EXE solutions. All solutions were homogenized at room temperature. After complete solubilization, 1 mL of counter-ion solution was added to 1 mL EXE solution directly in 2-mL Eppendorf tubes. The samples were first vortexed, followed by centrifugation at 21,000 g with a Centrifuge Fresco 21 (Thermo Fisher Scientific) at room temperature for 10 min. Supernatants were separated from the precipitate to quantify the non-complexed EXE fraction by HPLC and further calculate the precipitation efficiency as described below:

$$Precipitation\;efficiency=100-\left(100*\frac{{\left[EXE\right]}_{supernatant}}{{\left[EXE\right]}_{total}}\right).$$

Precipitates were freeze-dried (LyoBeta Mini, Telstar, Elancourt, France) using the following parameters: samples were frozen at −45 °C with a temperature ramp of 1 °C/min, then sublimated at −30 °C and 0.05 mbar for at least 24 h, and finally underwent desorption under maximum vacuum at −10 °C for 2 h. The final freeze-dried products were stored at −20 °C in a freezer. Each ratio of each counter-ion was tested in triplicate (three independent preparations of HIP).

Differences between the groups were analyzed using a two-way analysis of variance (ANOVA) with the counter-ion and ratio as factors. When significant effects were observed, multiple comparisons were performed using Sidak’s *post hoc* test to compare the two products at each ratio. A *p*-value <0.05 was considered statistically significant.

#### 2.2.3 Log P measurement

A total of 5 mg of EXE or selected HIP were added to 10 mL of n-octanol:water (1:1) mixture and incubated at room temperature under stirring for 24 h. The samples were centrifuged for 5 min at 5,000 rpm at room temperature using a Sorvall ST16 Centrifuge (Thermo Fisher Scientific). The amount of EXE in each phase was quantified using the method described above. The partition coefficient, Log P, was calculated as follows:

$$\mathrm{Log}\;P=\log\left(\frac{{\left[EXE\right]}_{octanol}}{{\left[EXE\right]}_{water}}\right).$$

#### 2.2.4 Solubility evaluation

To measure the solubility of EXE and EXE.DOC HIP in a selection of lipid excipients (see Table 1), each of the compound was placed in Eppendorf tubes to which liquid excipients were added to reach a minimal concentration of 10 mg/g (all excipients were saturated). Samples were vortexed after excipient addition and let under agitation at 2,500 rpm for 72 h at 25 °C using an orbital agitator (ThermoMixer F2.0, Eppendorf, Montesson, France). After 24 h, 48 h, and 72 h, samples were centrifuged at 2,800 g with a Centrifuge Fresco 21 (Thermo Fisher Scientific) at 25 °C for 30 min. The amount of EXE in the supernatant was quantified using the method described in Section 3.2.1.

C10 was dispersed under magnetic stirring in a selection of lipid excipients (see Table 1) applying four different concentrations: 12.5 mg/g, 25 mg/g, 50 mg/g, and 100 mg/g. Solubility was assessed visually after a stirring period of 30 min.

#### 2.2.5 LBF formulation design

Miscible binary and ternary mixtures of excipients were prepared based on solubility measurements. Their emulsifying properties were evaluated by dispersing 100 µL of placebo formulation in 100 mL of ultra-purified water at 37 °C under gentle magnetic stirring (50 rpm). Particle size dispersion was measured at T0 and 2 h after the dispersion by dynamic light scattering using a Zetasizer Nano ZS (Malvern, Palaiseau, France). Only transparent or translucent dispersions (including those showing bluish Tyndall effect) were analyzed, as required for reliable DLS measurements, while cloudy or turbid samples were excluded. Prior to analysis, samples were filtered on 1-µm pore-size filters to remove potential dust particles or external contaminants that could interfere with the light scattering measurement. This pore size was selected to avoid the removal of droplets within the expected measurement range (<500 nm). Measurements were performed in triplicate (three independent preparations).

#### 2.2.6 Protective effect on α-chymotrypsin-induced degradation

α-chymotrypsin was selected as a model for intestinal protease because it is the most active pancreatic enzyme against EXE (Sun et al., 2016). Since clear data on enzyme concentrations in human intestinal fluids were not available during method development, the α-chymotrypsin concentration was set to achieve complete degradation of EXE within a maximum of 1 h under the test conditions.

α-chymotrypsin solution was prepared at a concentration of 7.50 μg/mL in ultra-purified water. EXE- and EXE.DOC-loaded formulations were diluted in water to obtain a concentration of 300 μg/mL. For the degradation assay, 1 mL of the EXE solution was added to 8 mL of the α-chymotrypsin solution at room temperature. At predetermined time-points, 1-mL aliquots were withdrawn, and the enzymatic activity was immediately quenched by the addition of 132 µL of HCl 6.0 N. The EXE content in the samples were analyzed by HPLC.

All experiments were performed in triplicate for the control and for each formulation (three independent preparations). Statistical analysis was performed using two-way ANOVA, followed by Dunnett’s *post hoc* test to evaluate the protective effect of the formulations compared with that of the EXE solution control.

#### 2.2.7 Antioxidant selection

The oxidation (Epa) and reduction (Epc) potentials of EXE.Ac, EXE.DOC, and a range of antioxidants (ascorbic acid, BHA, BHT, DL α-tocopherol, and propyl gallate) were evaluated by cyclic voltammetry using an Electrochemical Impedance Analyzer (µ-Autolab Type III, Metrohm, Villebon, France). Tetrabutyl ammonium tetrafluoroborate solutions, i.e., electrolyte solutions, were prepared at 0.1 M in methanol and DMSO. API or antioxidants were added at a concentration of 0.5 mM to the electrolyte solutions. Voltammetric measurements were conducted between −200 and 1,600 mV, with a scan speed of 0.1 V/sec. Five successive measurements were conducted for each sample.

#### 2.2.8 Formulation stability

A short-term proof-of-concept stability evaluation was conducted to rapidly compare the intrinsic stability of the different formulation concepts under refrigerated and room-temperature conditions without the intent to comply with regulatory stability guidelines.

Ternary formulations (85% Labrafac™ MC60, 10% Kolliphor® RH40, and 5% propylene glycol) were prepared and loaded with 6 mg/g EXE, as EXE.Ac or EXE.DOC, and, eventually, C10 (20 mg/g). Formulations were either stored at 5 °C or at room temperature (25 °C) for 4 weeks. Every week, 200-µL aliquots were withdrawn, solubilized in methanol, and analyzed by HPLC-UV using the assay RS method to track the apparition of relative substances. For suspensions, samples were vortexed prior to the withdrawal of aliquots. Samples stored at 5 °C were let at 25 °C for 30 min under magnetic stirring and vortexed before the withdrawal of aliquots. Samples were injected in triplicate.

The effect of time and storage condition on EXE stability was analyzed using a two-way ANOVA. When significant effects were detected, multiple comparisons were performed using Dunnett’s *post hoc* test to compare T0 vs. T4W with both storage conditions.

#### 2.2.9 Enteric disintegration test of filled capsules

Size #0 bilayer capsules, Enprotect® or customized capsules, were filled with the formulations and closed. Enteric disintegration testing was performed in accordance with the USP 〈701〉 disintegration procedure for delayed-release tablets and capsules. Filled capsules were placed in the tubes of a USP type II disintegration Apparatus B (SOTAX DT2) maintained at 37.0 °C ± 0.5 °C. Capsules were first exposed to 720 mL HCl (0.1 N) for 1 h without sinkers or disks and subsequently transferred to 720 mL of phosphate buffer at pH 6.8 for 1 h without sinkers and with disks. Each test was performed on six capsules. To meet the acceptance criteria for delayed-release dosage forms, capsules were required to show no evidence of disintegration, cracking, or softening during the acid phase. Complete disintegration was expected to occur during the buffer stage in less than 1 h.

#### 2.2.10 Capsule manufacturing and characterization

Size #0 bilayer capsules with gelatin structural layer and HPMC-AS functional layer were prepared on the pilot scale capsule manufacturing equipment using Capsugel double-dipping patented technology (He et al., 2023).

Enteric properties of the customized capsules were evaluated by a 2-steps dissolution method using Apparatus type II (Sotax, Saint-Louis, France) and EMT as the acid-sensitive marker. Briefly, the capsules were filled with 100 mg of EMT pellets (corresponding to 20 ± 1 mg EMT) and closed. Each capsule was placed in 300 mL HCl 0.1 N at 37 °C and 100 rpm. After 2 h in the acid medium, 700 mL of 0.086 N dibasic sodium phosphate buffer (pH adjusted to 6.80 ± 0.05) was added to the vessel, and the test was maintained for an additional hour. During the buffer stage, samples were withdrawn at defined time-points and immediately stabilized by the addition of NaOH 0.25 N to avoid EMT degradation prior to analysis. The test was conducted on six capsules. The acceptance criteria were defined in accordance with the USP monograph for delayed-release esomeprazole capsules, which is no more than 10% of labeled amount of EMT dissolved after 2 h in acid stage and not less than 80% dissolved within 30 min in the buffer stage.

## 3 Results

### 3.1 Preparation and characterization of exenatide lipophilic salts

**FIGURE 2:**
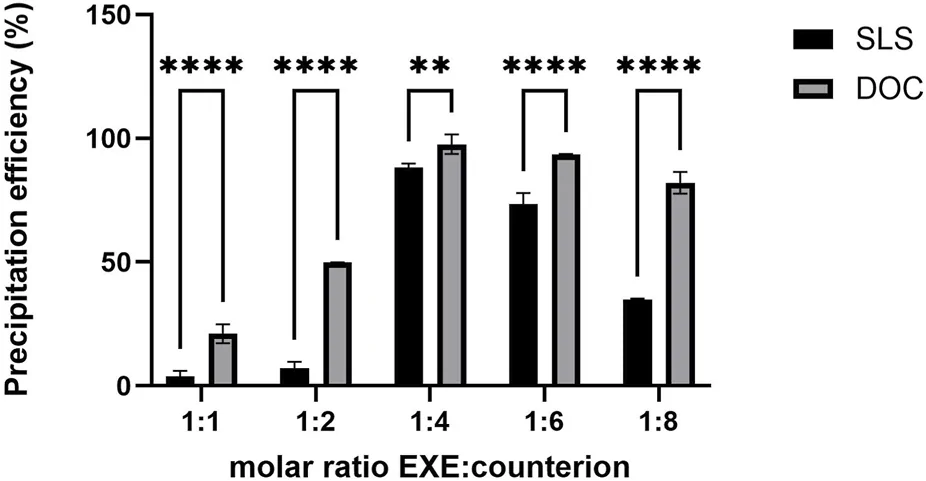
Precipitation efficiency of HIP formed with DOC and SLS as counter-ions; data are represented as the mean ± standard deviation (n = 3). Statistical significance is indicated as follows: *p < 0.05, **p < 0.01, ***p < 0.001, and ****p < 0.0001.

The precipitation efficiency was calculated by quantifying the unprecipitated concentration of EXE after the formation of HIP in the presence of different molar ratios of anionic surfactants. For DOC and SLS, the highest values were obtained for the molar ratio 1:4, 97.6% ± 4.0% and 88.2% ± 1.7%, respectively (Figure 2), with significantly higher values for DOC than for SLS. At this optimal ratio, the EXE content in the obtained EXE.DOC and EXE.SLS HIPs was 93% ± 10% and 95% ± 12% of the theoretical value, respectively, indicating that the residual excess of counter-ions in the HIPs was limited to less than 7%. Notably, intra-batch variability was substantially lower, as exemplified by EXE.DOC batches showing an EXE content of 95% ± 2%. The higher inter-batch variability was mainly attributed to difficulties in accurately weighing the electrostatic, freeze-dried HIP flakes stored in plastic Eppendorf tubes. In addition, the FT-IR scans confirm the pairing of counter-ions to EXE with the display of the specific bands of the counter-ions (Supplementary Figures 1, 2).

**FIGURE 3:**
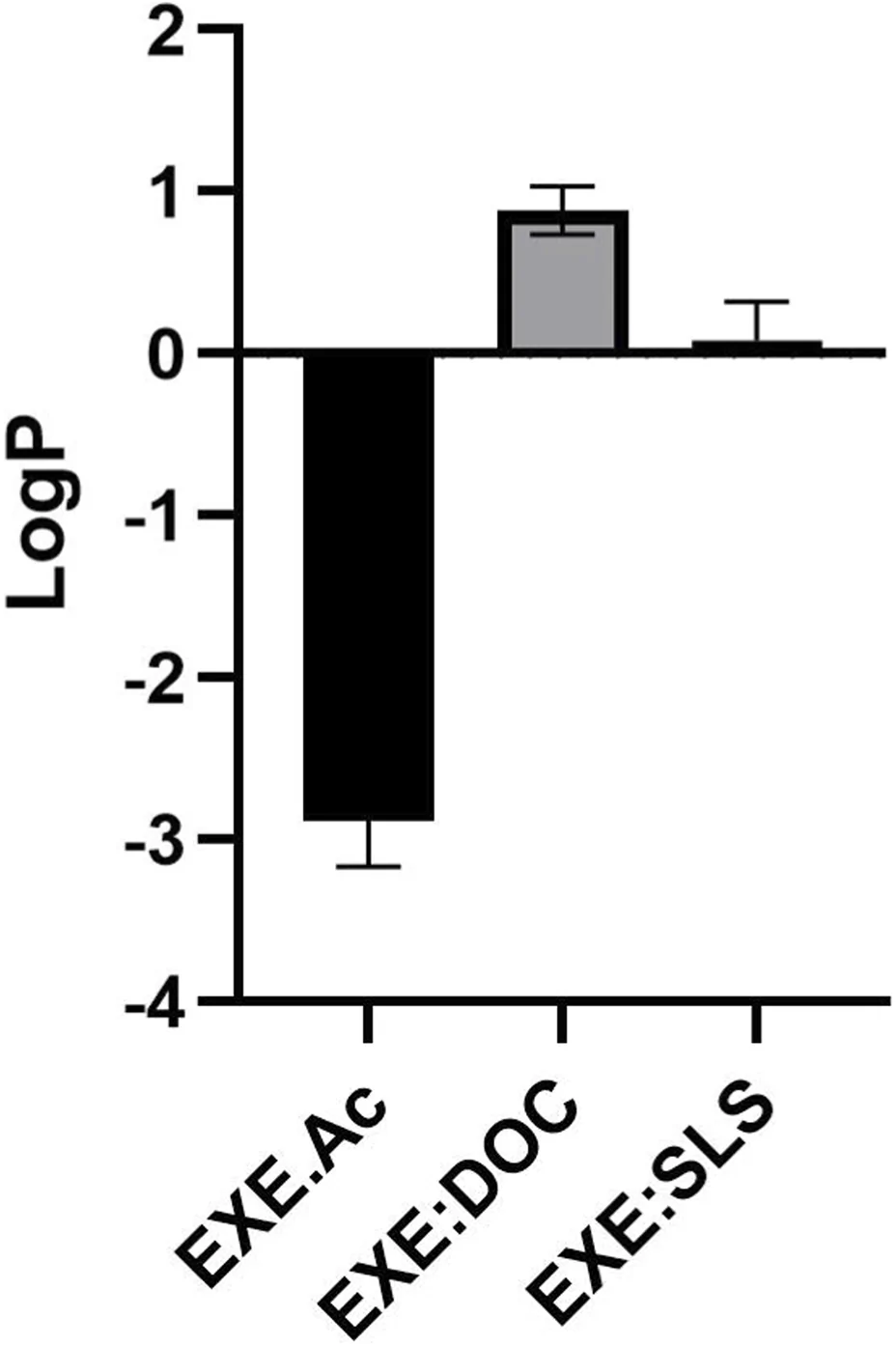
Log P of EXE.Ac, EXE:SLS (1:4), and EXE:DOC (1:4). Data are represented as the mean ± standard deviation (n = 3).

Log P measurements confirmed the ability of HIP to increase lipophilicity with a value of −2.9 ± 0.3 for EXE.Ac and values of 0.1 ± 0.2 and 0.9 ± 0.2 for EXE.SLS and EXE.DOC, at a 1:4 molar ratio, respectively (Figure 3 and Supplementary Table 1). EXE.DOC (1:4) was selected for the next stages of the formulation development process. To facilitate the reading, this lipophilic salt will be referred as HIP in the following sections of this article.

### 3.2 Formulation design

Solubility measurements of EXE.Ac and HIP in lipid excipients are depicted in Table 3. The results show limited solubility in lipid excipients for EXE.Ac, except in hydrophilic solvents. Modification of EXE lipophilicity by complexation with DOC facilitated its dissolution in lipid excipients, with the highest solubility measured in Labrafac™ MC60, Labrasol® ALF, and propylene glycol. However, for Labrasol® ALF and PEG 400, increasing concentrations of impurities were measured in the samples during the 72-h duration of the solubility evaluation. This could be caused by an interaction with PEG 400, which is also present in Labrasol® ALF, or with products of PEG degradation such as peroxides or formaldehydes (Jannin et al., 2014).

**TABLE 3:** Orientating solubility measurements of EXE.Ac, HIP, and C10 in lipid excipients.

Type	Excipients	EXE.Ac	HIP	C10
Oils	Maisine® CC	<LOQ	0.03	12.5
	Soybean oil	<LOQ	<LOQ	<12.5
Surfactants	Capryol® 90	<LOQ	0.02	<12.5
	Labrafac™ MC60	<LOQ	9.54	25
	Lauroglycol™ 90	<LOQ	<LOQ	<12.5
	Labrafil® M1944CS	0.7	<LOQ	<12.5
	Labrasol® ALF	<LOQ	5.47^a^	<12.5
	Kolliphor® ELP	<LOQ	<LOQ	<12.5
	Tween® 20	<LOQ	0.06	<12.5
	Tween® 80	<LOQ	<LOQ	<12.5
Solvents	PEG 400	<LOQ	2.28^a^	<12.5
	Propylene glycol	9.9	9.04	100
	Glycerin	5.2	0.89	<12.5

^a^ Increasing concentrations of relative substances were observed in samples through the solubility evaluation.

In parallel, solubility evaluation of C10 in the same selection of excipients demonstrated that the best solubilizer for the permeation enhancer were Labrafac™ MC60 and propylene glycol. Labrafac™ MC60 was, therefore, selected to be used as the main component of the LBF to maximize the solubilized API and C10 content in the final dosage form.

To obtain a self-emulsifying drug delivery system upon dispersion in gastro-intestinal fluids, Labrafac™ MC60 was combined with various surfactants. PSD of the formulations with Z-average below 600 nm are displayed in Table 4. Results show that poloxamers provided limited emulsifying properties, with a large amount (30%) needed to obtain particle sizes in the 200 nm range with very heterogeneous dispersion (PDI>0.4). On the contrary, with ethoxylated castor oil-based surfactants Kolliphor® ELP and Kolliphor® RH40, 10% of the surfactants were sufficient to obtain monodispersed formulations with low PSD (149.3 ± 0.9 nm and 117.2 ± 0.6 nm, respectively).

**TABLE 4:** Particle size distribution of the prototype formulations (placebo) upon dispersion (1:1,000) in water at 37 °C, n = 3; Labrafac^™^MC60 = MC60, Kolliphor^®^ ELP = KELP, Kolliphor^®^ RH40 = RH40, polysorbate 20 = PS20, polysorbate 80 = PS80, and propylene glycol = PG.

Formulations	Lipid excipients (%, w/w)						Z-average (nm)	PDI
	MC60	KELP	RH40	PS20	PS80	PG		
F1	90	10	—	—	—	—	149.3 ± 0.9	0.192 ± 0.080
F2	85	15	—	—	—	—	69.2 ± 1.0	0.095 ± 0.050
F3	80	20	—	—	—	—	49.8 ± 0.4	0.043 ± 0.080
F4	70	30	—	—	—	—	127.8 ± 2.5	0.534 ± 0.108
F5	90	—	10	—	—	—	117.2 ± 0.6	0.082 ± 0.016
F6	85	—	15	—	—	—	72.8 ± 0.9	0.099 ± 0.005
F7	80	—	20	—	—	—	40.7 ± 0.1	0.038 ± 0.004
F8	70	—	30	—	—	—	32.7 ± 0.2	0.171 ± 0.004
F9	85	—	10	—	—	5	106.2 ± 0.9	0.085 ± 0.030
F10	70	—	—	30	—	—	247.2 ± 2.6	0.410 ± 0.016
F11	70	—	—	—	30	—	329.1 ± 6.1	0.643 ± 0.028

The addition of small quantities of solvent in lipid formulations can boost API solubilization (Pouton, 2000) and facilitate emulsification (Jörgensen et al., 2020). Therefore 5% of propylene glycol was integrated in the formulation F5, which exhibited the smallest Z-average value with 10% surfactant. The particle size of the formulation thus obtained, F9, was slightly decreased, with a final Z-average of 106.2 ± 0.9 and a PDI of 0.085 ± 0.030.

The stability of emulsified F5 and F9 in water at 37 °C was confirmed over 2 h (Table 5).

**TABLE 5:** PSD evolution of formulations F5 and F9 upon dispersion in water (1:1,000) at 37 °C, n = 3.

Formulations	Time (h)	Z-average (nm)	PDI
F5	0	117.2 ± 0.6	0.082 ± 0.016
	2	118.2 ± 1.0	0.088 ± 0.080
F9	0	106.2 ± 0.9	0.085 ± 0.030
	2	108.3 ± 0.7	0.083 ± 0.010

### 3.3 Formulation characterization

A complementary solubility study was conducted to further differentiate F5 and F9 based on their solubilization capacity of EXE.Ac and HIP. The results, depicted in Table 6, confirm the increased solubilization capacity of HIP compared to that of the EXE.Ac form. The solubility results also show the benefits of the addition of propylene glycol to increase the formulation solubilization capacity of both EXE.Ac and HIP. Finally, the incorporation of C10 in the formulations shows an important increase in the solubility of EXE regardless of the presence of propylene glycol and the EXE salt form. F9 was selected for the following steps of the development of the EXE oral-dosage form. To avoid premature drug release from the formulation upon its dilution in the aqueous fluids of the GI tract (Williams et al., 2012a; 2012b), the final concentration of EXE as HIP in the formulations was set at 80% of the measured solubility, which was 6 mg/g.

**TABLE 6:** EXE solubility evaluation in F5 and F9 as the function of the EXE form and the presence of C10.

Formulations	Formulation composition (%, w/w)			C10 (20 mg/g)	EXE form	EXE solubility (mg/mL)
	MC60	RH40	Propylene glycol			
F5	90	10	​	No	EXE.Ac	0.074 ± 0.010
F5	90	10	​	No	HIP	4.887 ± 0.185
F5 C10	90	10	​	Yes	EXE.Ac	0.096 ± 0.020
F5 C10	90	10	​	Yes	HIP	7.709 ± 0.272
F9	85	10	5	No	EXE.Ac	0.112 ± 0.004
F9	85	10	5	No	HIP	7.283 ± 0.346
F9 C10	85	10	5	Yes	EXE.Ac	1.436 ± 0.007
F9 C10	85	10	5	Yes	HIP	7.635 ± 0.171

### 3.4 *In vitro* proteolysis

**FIGURE 4:**
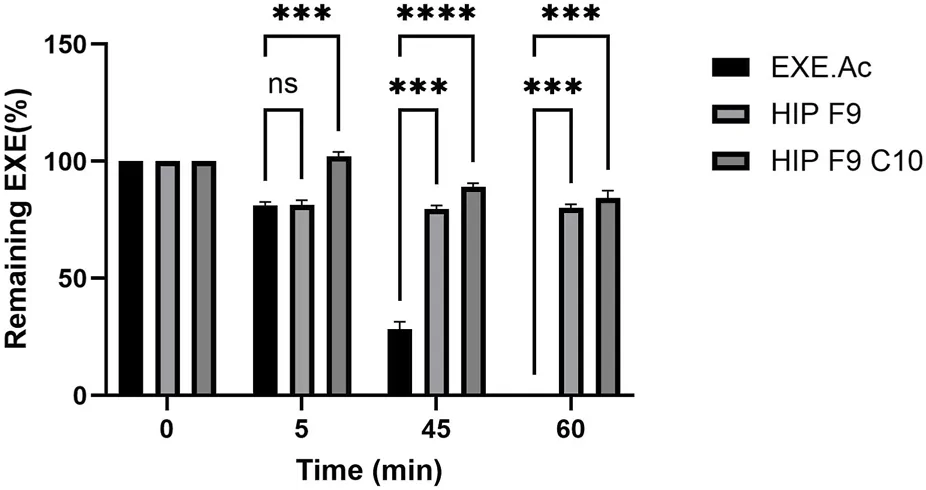
Degradation profiles of EXE in the presence of α-chymotrypsin. Data are expressed as the mean ± standard deviation (n = 3). Statistical significance is indicated as follows: *p < 0.05, **p < 0.01, ***p < 0.001, and ****p < 0.0001.

The results presented in Figure 4 confirm the rapid degradation of EXE in the presence of α-chymotrypsin. However, when EXE is dissolved in LBF as HIP, degradation is significantly limited, with approximately 80% of the API recovered after 60 min in the presence of the protease. Inclusion of C10 in the formulation appears to positively impact the protective effect in the first five minutes in the presence of α-chymotrypsin. However 60 min after the initiation of the test, the remaining EXE in the test medium was in the same range regardless of whether C10 was present in the formulation (84% ± 3%) or absent (80% ± 1%). The degradation rates between the two formulation prototypes were not significantly different.

### 3.5 Stability study

The sensitivity of EXE to oxidation was measured by cyclic voltammetry. No oxidoreduction potentials could be measured; indicating that EXE is not prone to electrochemical oxidation at the electrode surface under tightly controlled experimental conditions (Supplementary Figures 3–8). However, based on the EXE structure, some amino acid residues such as methionine 14 and tryptophan 25 could be susceptible to oxidation in lipid matrices (peroxides, aldehydes, or metal ions; Jannin et al., 2014). The cyclic voltammetry method cannot be used to determine the most efficient antioxidant to be added into the formulation to protect EXE from oxidation. However, the use of fresh lipid excipient samples and control of the process and storage conditions to avoid exposure to light and dioxygen are recommended to mitigate risk.

**FIGURE 5:**
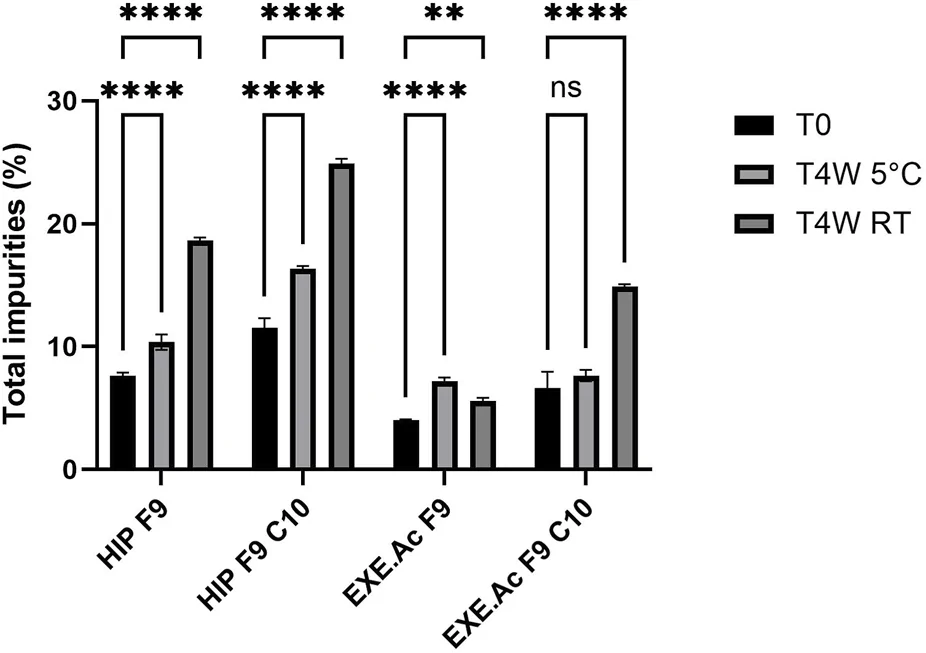
Evolution of the EXE impurity content in the formulations depending on the formulation composition, EXE salt type, and storage conditions over 4 weeks. Data are represented as the mean ± standard deviation (n = 3). Statistical significance is indicated as follows: *p < 0.05, **p < 0.01, ***p < 0.001, and ****p < 0.0001.

A short EXE stability evaluation in the designed formulations was performed at 5 °C and at room temperature over 4 weeks. The evolution of the impurity content over time is reported in Figure 5 (see also Supplementary Figure 9; Tables 2 and 3 for detailed evolution of major impurities). A significant increase in the impurity content was observed for all formulations, except when EXE was present as EXE.Ac in the suspension in the formulation with C10 and stored at 5 °C. With the formulation (without C10), there was no statistical difference in the formation of impurities between EXE in solution (HIP) and in suspension (EXE.Ac) (*p* > 0.05). However, at room temperature, significantly less impurity formation was observed with the suspension of EXE.Ac than with the solution (*p* < 0.01). Finally, the addition of C10 accelerated the degradation of EXE at room temperature (*p* < 0.01).

### 3.6 Disintegration test with the Enprotect® capsule

**FIGURE 6:**
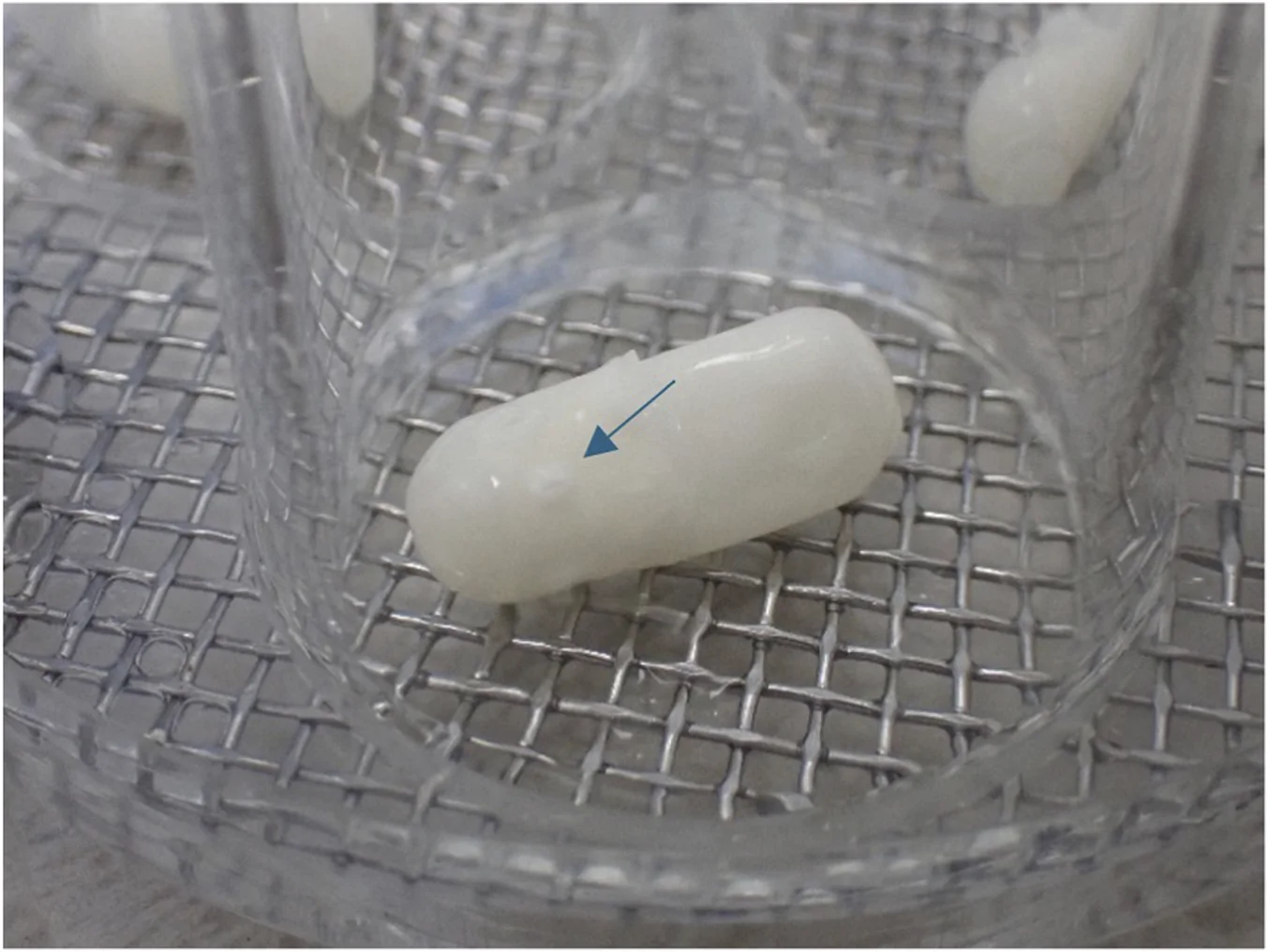
Exemplar picture of a Capsugel® Enprotect® capsule filled with placebo F9 after 1 h in HCl 0.1 N, with an arrow pointing at the hole formed during the test.

A disintegration test performed on standard size #0 white Enprotect® capsules filled with placebo F9 formulation highlighted the formation of holes in the caps of three out of six tested capsules within 30 min after the initiation of the test in acidic pH (Figure 6). The dosage forms were, therefore, not compliant with USP pharmacopeia.

### 3.7 Manufacturing and characterization of customized enteric capsules

**FIGURE 7:**
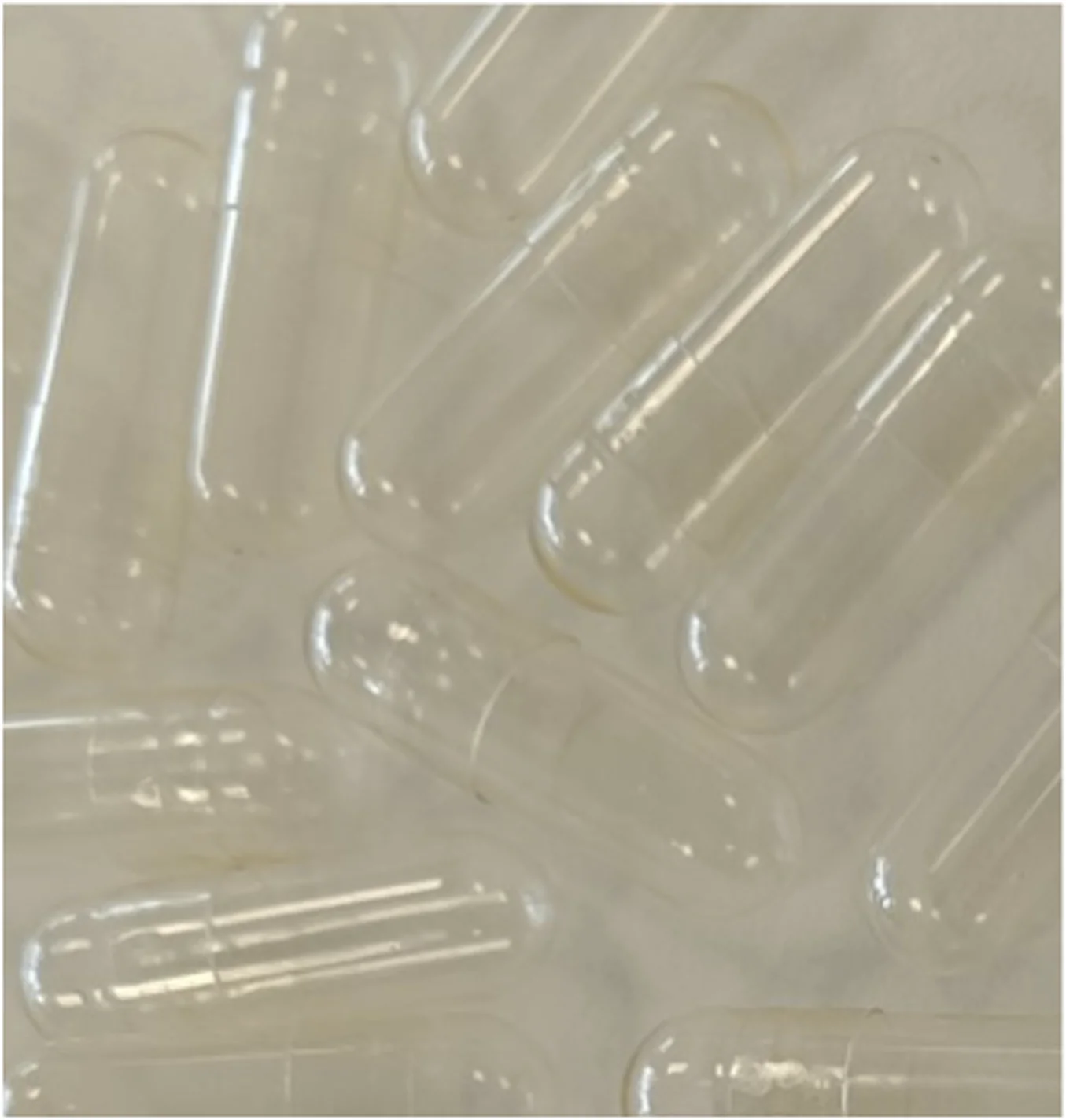
Exemplar images of customized size #0 transparent bilayer gelatin/HPMC-AS capsules.

Customized transparent size #0 double-layer capsules with an inner gelatin structural layer and external HPMC-AS layer were successfully manufactured on pilot-scale capsule-manufacturing equipment. Capsules presented conforming aspect with no visible defaults (Figure 7). The customized capsule parts measured 18.0 ± 0.1 mm (body, n = 20) and 10.7 ± 0.9 mm (cap, n = 20), which is in a similar range as the standard size #0 Capsugel® Enprotect® capsules with the capsule body length of 18.44 mm and cap length of 10.72 mm (with a tolerance of ±0.46 mm).

**FIGURE 8:**
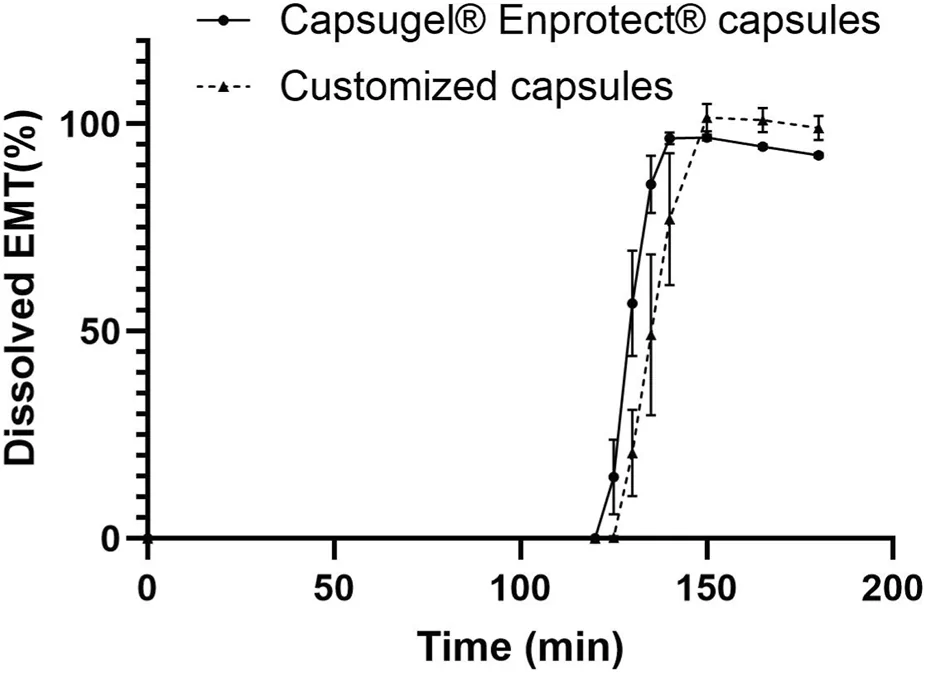
Dissolution profile of EMT pellets encapsulated in a size #0 Capsugel® Enprotect® capsule and in customized size #0 transparent gelatin/HPMC-AS capsules during 2 h in HCl 0.1 N followed by 1 h in buffer at pH 6.8. Data are represented as the mean ± standard deviation (n = 6).

Enteric properties of the customized capsules were evaluated by performing a dissolution test on capsules filled with an acid-sensitive small molecule (EMT). The dissolution profile, depicted in Figure 8, is very similar to the dissolution profile obtained with Capsugel® Enprotect® capsules. It shows that no EMT was released from the capsules during the 2 h in an acid medium, while a swift release of the API was observed once the medium was switched for buffer pH 6.8. The complete encapsulated dose was released within 30 min after changing the medium, and fully recovered, confirming that the customized capsules were compliant with USP monographs for delayed-release dosage forms.

### 3.8 Enteric properties of filled customized enteric capsules

**FIGURE 9:**
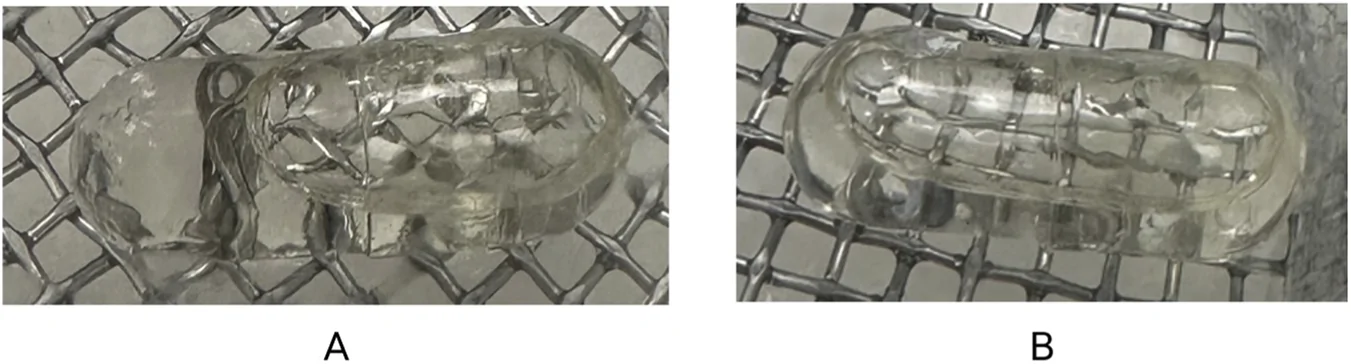
Exemplar images of customized size #0 gelatin/HPMC-AS bilayer capsules filled with **(A)** placebo F9 (including 20 mg/g C10) and **(B)** F9 loaded with HIP (EXE dose 6 mg/g) and 20 mg/g C10 after 1 h in HCl 0.1 N

A disintegration test performed on customized capsules filled with placebo F9, including C10, showed no opening or degradation of the capsule shell integrity after 1 h in HCl 0.1N (Figure 9). All capsules (n = 6) showed fast opening within 5 min upon switching the medium to phosphate buffer with pH 6.8. The same observations were made when the gelatin-based enteric capsules were filled with the same formulation in which 6 mg/g EXE were dissolved in lipidized form (HIP).

## 4 Discussion

### 4.1 HIP formation and lipophilicity enhancement

This study demonstrated the successful formation of HIP between EXE and 2 IID listed anionic surfactants (DOC and SLS) to increase peptide lipophilicity. For both counter-ions, the optimal molar ratio of 1:4 (EXE:anionic surfactant) was observed to maximize lipophilic salt formation. This result is consistent with the presence of four ionizable amino acids in EXE (lysine, 2 histidine, and arginine) under acidic conditions, enabling complete charge neutralization in the presence of the four anionic charges provided by the single-charged counter parts, as previously reported by Ristroph and Prud’homme (2019). The highest lipophilicity increase was obtained with DOC with a log P increase of approximately 3 units, which indicates that EXE affinity for lipophilic environment was multiplied by a factor 1,000. The superior performance of DOC can be explained by its branched alkyl structure combined with sulfosuccinate moiety, which confers greater hydrophobic character and stronger van der Waals interactions with the peptide. This observation aligns with results from Menzel et al., who reported similar log P shifts upon complexation of EXE with DOC, confirming that the use of docusate as a counter-ion is particularly effective for peptide lipidization (Griesser et al., 2017; Menzel et al., 2018).

### 4.2 HIP formation favors dissolution in lipid excipients and the design of LBF with features to promote intestinal absorption

HIP formation markedly improved EXE solubility in medium-chain-based lipid excipients, particularly in Labrafac™ MC60, compared to that of EXE.Ac. This excipient, glycerol monocaprylocaprate, is known to act both as a solubilizer and a mild intestinal permeation enhancer, justifying its selection as the main lipid phase of the LBF (McCartney et al., 2024).

Among the tested surfactants, Kolliphor® RH40 allowed the formation of stable emulsions at low concentration (10%), with monodispersed droplets measuring approximately 100 nm (formulation F9). The addition of a small fraction of propylene glycol (5%) further enhanced the solubilization capacity and reduced the particle size, while preserving the emulsion stability over time. This synergistic effect can be attributed to the co-solvent’s ability to modulate interfacial tension and facilitate surfactant packing at the oil–water interface (Jörgensen et al., 2020). The final particle size, approximately 110 nm, should facilitate the diffusion of oily droplets across the intestinal mucus and facilitate peptide transportation to the epithelial border (Griesser et al., 2018).

C10 was incorporated at 20 mg/g of the formulation to maintain the lipid-based formulation in a homogeneous liquid state and facilitate the scalability of the encapsulation process. Inclusion of this medium-chain fatty acid further enhanced EXE solubilization independently of the physical form of the peptide. C10 is a well-documented PE that can induce transient epithelial membrane fluidization, complementing the effect of Labrafac™MC60 (Brayden et al., 2015). When combined with enteric capsule delivery, this strategy enables coordinated intestinal release of both the peptide and permeation enhancers while limiting the overall enhancer exposure.

The final formulation was designed at 80% of the experimentally determined solubility of EXE, corresponding to a drug concentration of 6 mg/g. This concentration delivers doses of 2.5 mg, 3.3 mg, and 4.7 mg when filled into size #1, #0, and #00 capsules, respectively. The final compositions of the prototypes are summarized in Table 7.

**TABLE 7:** Final composition of enteric capsules filled with design formulation.

Formulation components	Size 00 capsule	Size 0 capsule	Size 1 capsule
Capsule volume (mL)	0.91	0.68	0.50
Available volume (mL)	0.79	0.55	0.42
Labrafac^TM^ MC60 (g)	0.67	0.47	0.36
Kolliphor® RH40 (g)	0.08	0.06	0.04
Propylene glycol (g)	0.04	0.03	0.02
Exenatide HIP (mg)	6.68	4.65	3.55
Corresponding EXE (mg)	4.74	3.30	2.52
Sodium caprate (mg)	15.8	11.0	8.4

### 4.3 Peptide dissolution in LBF enables protection from enzymatic degradation

*In vitro* proteolysis assays confirmed the protective effect of the LBF containing HIP. While unformulated EXE underwent complete degradation by α-chymotrypsin within minutes, approximately 80% of the peptide remained intact after 60 min when incorporated as HIP in the LBF. This can be attributed to the shielding effect of the lipid matrix, reducing peptide exposure to enzyme with the higher lipophilic environment (Hetényi et al., 2017).

The observed partial benefit of C10 in the early stage of the proteolysis assay could be explained either by the increased solubility of the HIP in the LBF containing C10 or by a transient interaction of C10 with the protease. These results reinforce the potential of the combination of HIP and LBF as a protection and absorption-promoting strategy.

### 4.4 Stability evaluation

Although this short-term assessment was performed as a proof-of-concept rather than a regulatory stability study, it provides important mechanistic insights into how the formulation state of the peptide (dissolved versus suspended) influences the degradation behavior, which is highly relevant for early formulation screening and strategy selection.

Evaluation of EXE stability in different formulations indicates accelerated degradation of the peptide at room temperature compared to that with storage at 5 °C, particularly when EXE was incorporated as HIP. This can be attributed to the higher reactivity of molecules in a solubilized state and is further supported by the lower impurity levels observed when EXE was incorporated as a suspension of EXE.Ac. In addition, Labrafac™ MC60, the main component of the formulation, is a glycerol monocaprylocaprate known to recrystallize at 5 °C. Although no solid particles were observed in the formulation upon aliquoting the stability assays, the complete API solubility may not have been recovered after formulation recrystallization.

The results obtained for formulations containing C10 are consistent with these observations. Enhanced EXE degradation was observed, aligning with the increased solubility of the API in the presence of C10. These results raise questions about the benefit of solubilizing the peptide in the LBF. This formulation strategy was originally considered for a gastric delivery, where a prolonged protection of the peptide is required until peptide absorption, either in the stomach or in the intestine. However, when combined with an enteric delivery system, LBF would be released under conditions with limited gastrointestinal fluids, leading to higher local API and lipid concentrations. This would promote co-localization of the API with high levels of permeation enhancer, rendering the pre-requisite of complete API solubilization less critical.

### 4.5 Capsule compatibility and enteric protection

The initial disintegration testing of standard Capsugel® Enprotect® capsules filled with the placebo formulation revealed a loss of capsule integrity in acidic environments. The phenomenon was attributed to incompatibility between the capsules’ HPMC inner layer and the lipid formulation.

To overcome this limitation, customized bilayer capsules featuring a gelatin structural inner layer and an HPMC-AS enteric outer layer were developed using the same double-dipping technology. Capsule modification maintained dimensional alignment with standard size #0 capsules, thus ensuring scalability for industrial filling processes. Dissolution testing with an acid-sensitive model API confirmed complete compliance with the USP criteria for delayed-release dosage forms: no capsule opening occurred during 2 h in 0.1 N HCl, followed by rapid and complete opening within minutes after transfer to pH 6.8 phosphate buffer. The disintegration test performed with the customized capsules and the designed formulation (F9 HIP C10) showed that the enteric properties of the capsule were maintained in the presence of the formulation. These results indicate that replacing the inner layer polymer from HPMC to gelatin enabled the compatibility with the formulation while maintaining the enteric properties. It highlights the versatility of double-dipping technology to manufacture functional capsules tailored to industry-specific needs for controlled-release delivery.

Beyond preserving gastro-resistance, the rapid capsule opening observed at intestinal pH supports the proposed mechanism of coordinated intestinal release of the peptide and permeation enhancers. Upon exposure to pH 6.8, the capsule releases the lipid formulation within minutes, enabling co-localization of EXE-HIP, sodium caprate, and Labrafac™ MC60 in the relatively small fluid volume of the small intestine (Mudie et al., 2014). This rapid dispersion is expected to limit premature dilution and potential displacement of the peptide from lipid droplets while maintaining the proximity between the API and permeation enhancers at the absorption site. Together with the previously demonstrated shielding effect of lipid droplets against proteolytic enzymes, this behavior supports a two-tier protection strategy in which the enteric capsule protects the formulation during gastric transit, while the lipid formulation contributes to peptide protection and localized delivery in the intestinal environment.

### 4.6 Limitations and future work

Although the present study demonstrates a coherent proof-of-concept for the oral delivery of EXE using HIP-loaded LBF with high potential for mucus and epithelial permeability incorporated into customized enteric capsules, several aspects require further analysis. Extended stability studies under various conditions should be conducted to elucidate the extent of EXE degradation depending of the physical state of the API, namely, solution (HIP) vs. suspension (EXE.Ac). Long-term compatibility studies between the formulation and the customized enteric capsule should also be conducted to assess the retention of the enteric properties upon storage. Finally, while the current study demonstrates *in vitro* protection and solubilization of EXE, these results provide a foundation for future *in vivo* pharmacokinetic and pharmacodynamic studies to determine whether these benefits translate into improved bioavailability and therapeutic efficacy.

### 4.7 Implications for oral peptide delivery

This work highlights the potential of combining the HIP formation with LBF technology and customized enteric capsule systems to address the multifaceted challenges of oral peptide delivery. The strategy allows formulation of therapeutic peptide doses in the milligram range, enhances solubility and enzymatic stability, and integrates permeation enhancers within a scalable dosage form. Compared to the existing oral peptide products such as Mycapssa®, a capsule that is enterically coated post-filling, this platform offers a simpler and potentially more versatile formulation pathway that is adaptable to a range of peptide APIs.

## 5 Conclusion

In this study, a lipid-based formulation of a GLP-1 peptide was successfully designed, achieving an encapsulation capacity of up to 3.3 mg solubilized EXE in the size #0 capsule. The formulation demonstrated strong protection against enzymatic degradation through peptide sequestration in lipid droplets and showed high potential for intestinal permeation with the inclusion of permeation enhancers. Simultaneously, a customized capsule was engineered to preserve gastro-resistance and enable targeted intestinal release of the formulation.

These findings highlight a proof-of-concept oral delivery platform combining HIP chemistry, lipid-based formulation, and ready-to-use enteric capsules. The approach provides a two-tier protection mechanism, a capsule shell against gastric conditions and lipid droplets against intestinal proteases, while minimizing premature gastric dilution and supporting the coordinated release of both the peptide and permeation enhancers at the absorption site. This strategy offers a scalable and mechanistically guided framework that may inform the future development of orally delivered peptide therapeutics as a viable alternative to parenteral administration.

## Data Availability

The original contributions presented in the study are included in the article/Supplementary Material; further inquiries can be directed to the corresponding author.
