# Correction: A two-tier protection strategy for oral delivery of GLP-1 peptides: lipid-based formulation combined with enteric capsules

**DOI:** 10.3389/fddev.2026.1973549

**Published:** 2026-09-15

**Authors:** Camille Dumont, Vanessa Gonzalez, Paula Klatt, Sandrine Picco, Delphine Nombret, Marine Agisson, Euengla Uku, Thibault Saurel, Vincent Jannin

**Affiliations:** Capsugel France SAS, Colmar, France

**Keywords:** customized capsule, enteric delivery, lipid-based formulations, oral peptide delivery, permeation enhancer

In the abstract, line 12, there is an inversion on the log P values of the EXE-HIP and EXE, it currently reads “*(log P = −2.9 ± 0.3 vs. 0.9 ± 0.2 for EXE)”*. This has been corrected to read: “*(log P = 0.9 ± 0.2 vs. −2.9 ± 0.3 for EXE)”*.

The original version of this article has been updated.

