# Supplementary material for: A two-tier protection strategy for oral delivery of GLP-1 peptides: lipid-based formulation combined with enteric capsules
Source: Front Drug Deliv. 2026 May 22;6:1770094. doi: 10.3389/fddev.2026.1770094 (PMC13236653; doi:10.3389/fddev.2026.1770094)
Supplement: Supplementary file 1 [file Supplementaryfile1.docx]

A two-tier protection strategy for oral delivery of GLP-1 peptides:

lipid-based formulation combined with enteric capsules

Camille Dumont^1^, Vanessa Gonzalez^1^, Paula Klatt^1^, Sandrine Picco^1^, Delphine Nombret^1^, Marine Agisson^1^, Euengla Uku^1^, Thibault Saurel^1^, Vincent Jannin^1^

^1^ Capsugel France SAS – Lonza, Colmar, France

Supplementary Material

**Supplementary Table 1**: Detailed Log P calculation of EXE.Ac, EXE:DOC (1:4) and EXE:SLS (1:4)

| **EXE.Ac** | | | | |
| --- | --- | --- | --- | --- |
| **Sample** | **API weight (mg)** | **EXE in Water (µg/mL)** | **EXE in Octanol (in µg/mL)** | **log P** |
| 1 | 5.033 | 980.703 | 0.809 | -3.08 |
| 2 | 5.060 | 1003.651 | 0.980 | -3.01 |
| 3 | 5.033 | 1000.809 | 2.851 | -2.55 |
| **Mean** | - | 995.054 | 1.547 | **-2.88** |
| **Std Dev** | - | 12.510 | 1.133 | **0.29** |
| **EXE:DOC (1:4)** | | | | |
| **Sample** | **API weight (mg)** | **EXE in Water (µg/mL)** | **EXE in Octanol (in µg/mL)** | **log P** |
| 1 | 5.426 | 1.038 | 10.210 | 0.99 |
| 2 | 5.085 | 2.760 | 16.461 | 0.78 |
| 3 | 5.676 | <LOQ | 14.067 | - |
| **Mean** | - | 1.899 | 13.579 | **0.88** |
| **Std Dev** | - | 1.218 | 3.154 | **0.15** |
| **EXE:SLS (1:4)** | | | | |
| **Sample** | **API weight (mg)** | **EXE in Water (µg/mL)** | **EXE in Octanol (in µg/mL)** | **log P** |
| 1 | 5.445 | 1.393 | 3.101 | 0.35 |
| 2 | 5.873 | 2.137 | 1.643 | -0.11 |
| 3 | 5.781 | 4.103 | 4.107 | 0.00 |
| **Mean** | - | 2.544 | 2.950 | **0.12** |
| **Std Dev** | - | 1.400 | 1.239 | **0.24** |

**Fourier Transform-Infrared Spectroscopy**

Measurements were performed using a Fourier Transform Infrared (FTIR) spectrophotometer (Nicolet 6700, Thermo Scientific, Oxford, UK). FTIR spectra were obtained in transmission mode from 4000 to 500 cm^-1^ at a 4 cm^-1^ resolution, and with an averaged 32 scans for background and each sample.

**Supplementary Figure 1**: FTIR spectra of exenatide acetate (blue), exenatide docusate HIP (purple) and sodium docusate (red), red arrows pointing at the characteristic bands representing the S=O stretching vibration, present on DOC and HIP EXE:DOC while absent from EXE.Ac


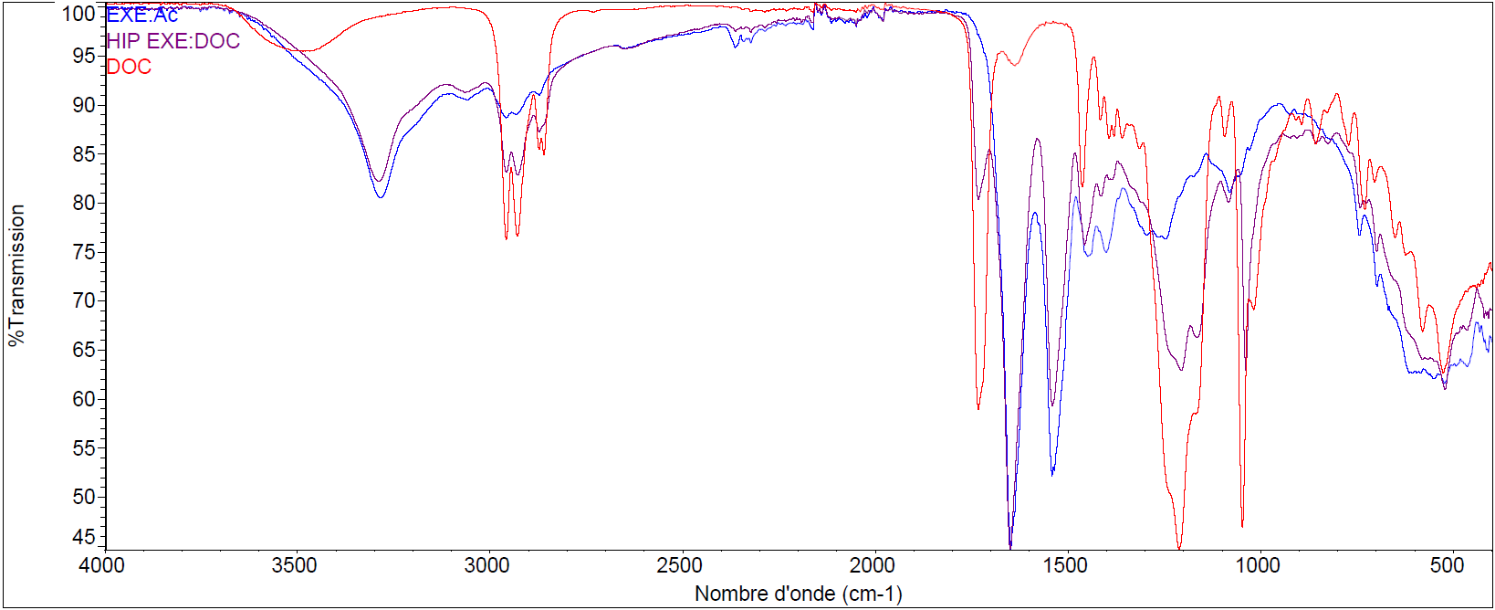


**Supplementary Figure 2**: FTIR spectra of exenatide acetate (blue), exenatide lauryl sulfate HIP (purple) and sodium lauryl sulfate (red); red arrows pointing at the characteristic bands representing the S=O stretching vibration, present on SLS and HIP EXE:SLS while absent from EXE.Ac


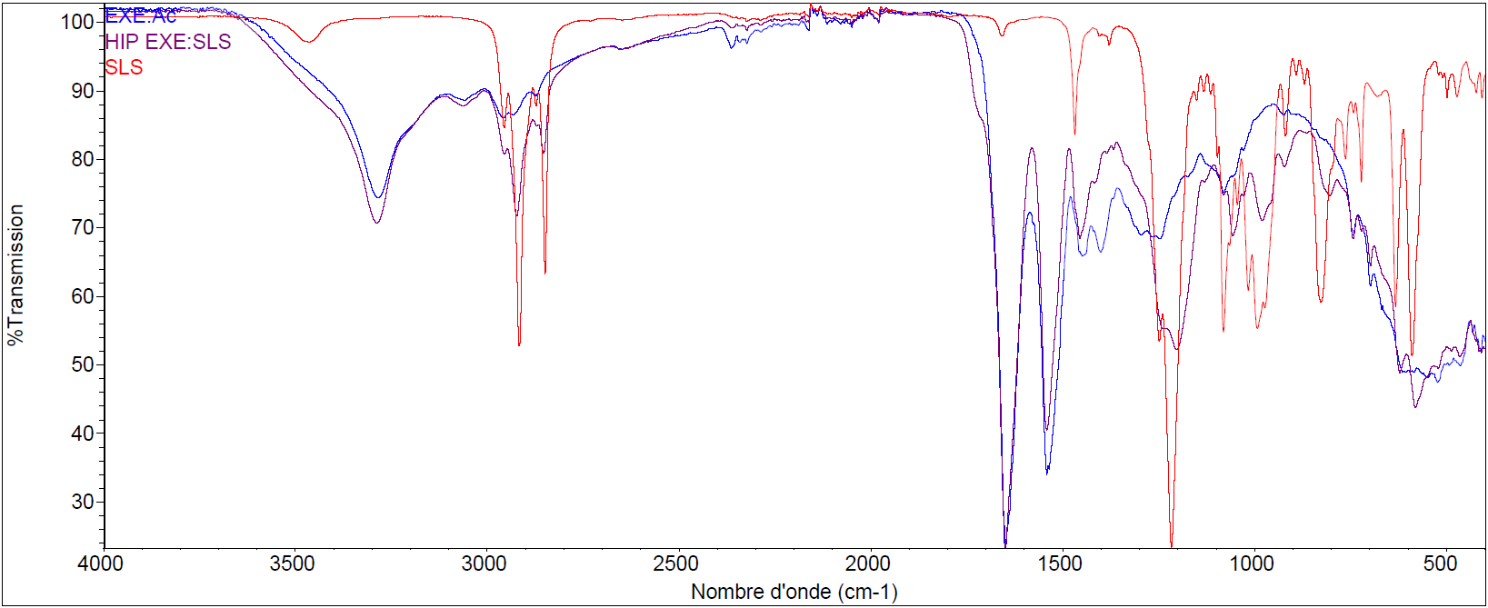


**
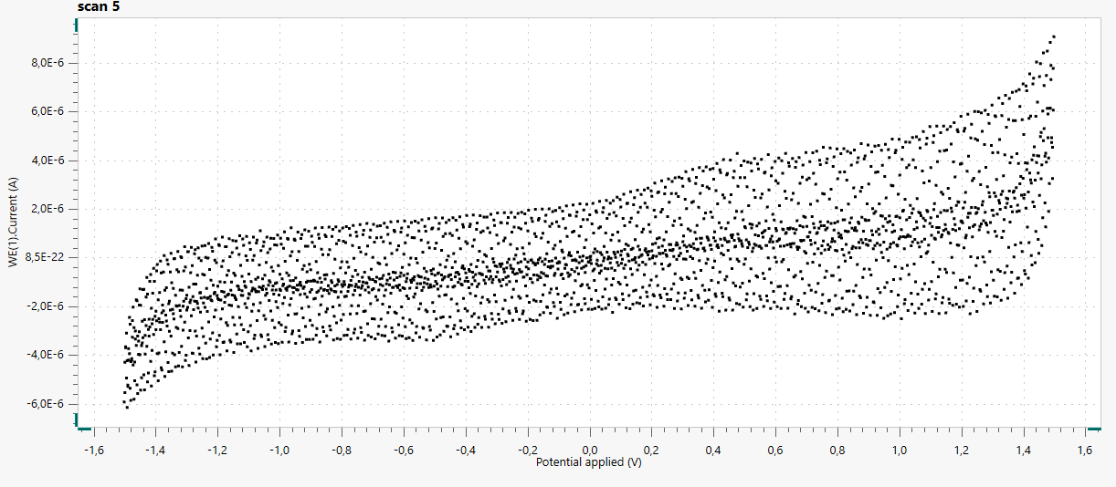
**

**Supplementary Figure 3.** Cyclic voltammogram of Exenatide in methanol.

**
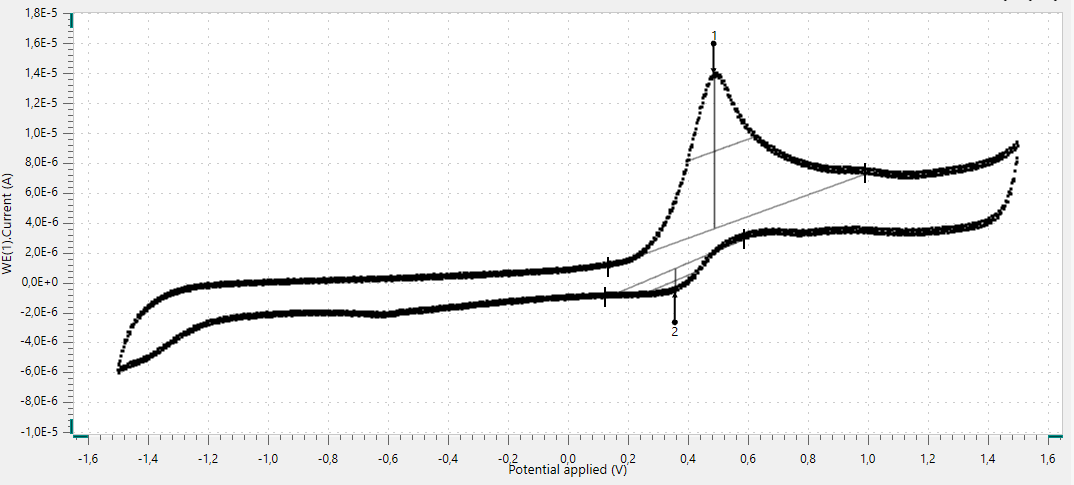
**

**Supplementary Figure 4.** Cyclic voltammogram of ascorbyl palmitate in methanol.

**
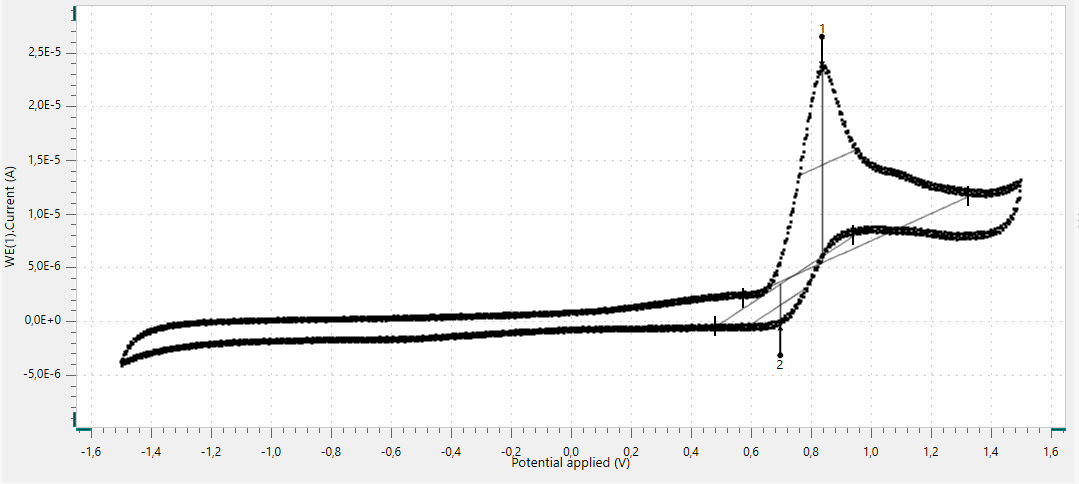
**

**Supplementary Figure 5:** Cyclic voltammogram of butylated hydroxyanisole (BHA) in methanol.

**
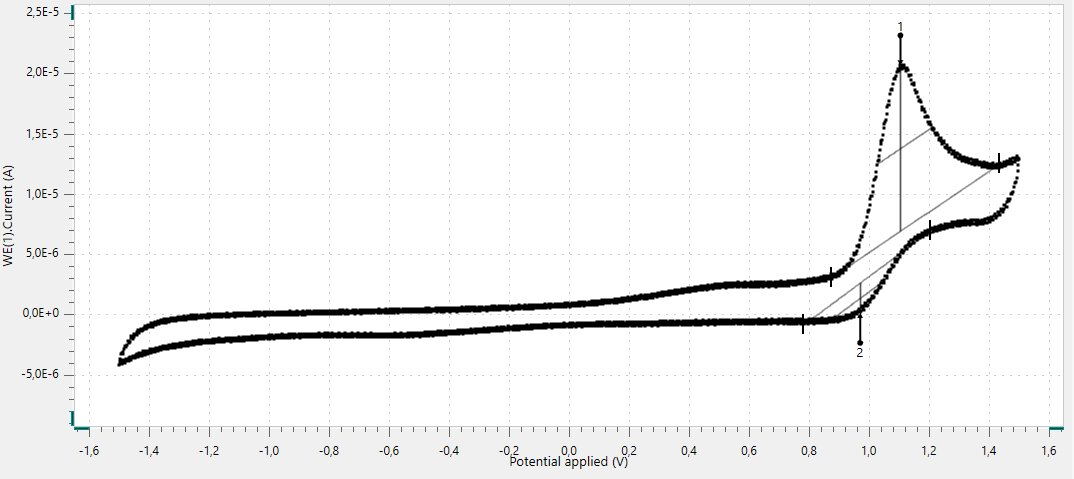
**

**Supplementary Figure 6:** Cyclic voltammogram of butylated hydroxytoluene (BHT) in methanol.

**
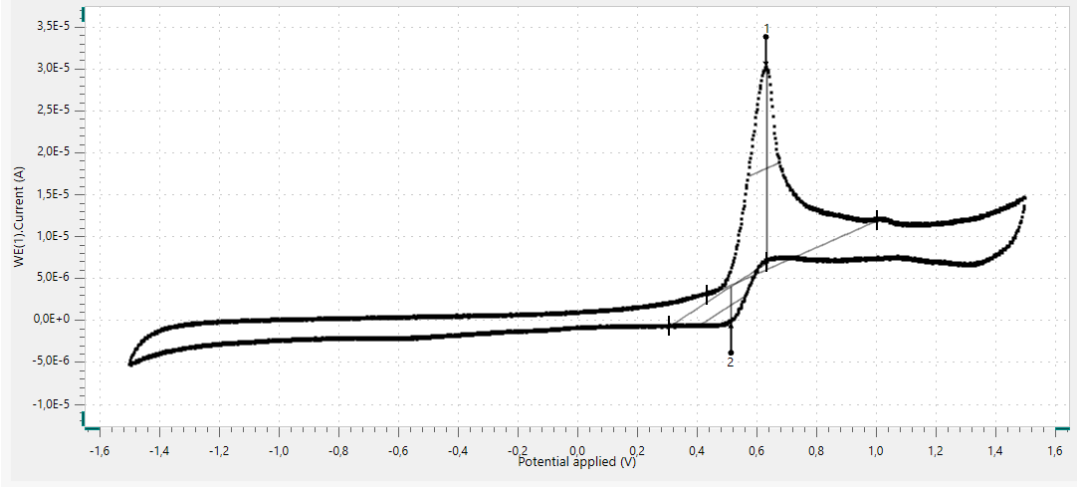
**

**Supplementary Figure 7:** Cyclic voltammogram of DL-α-tocopherol in methanol.

**
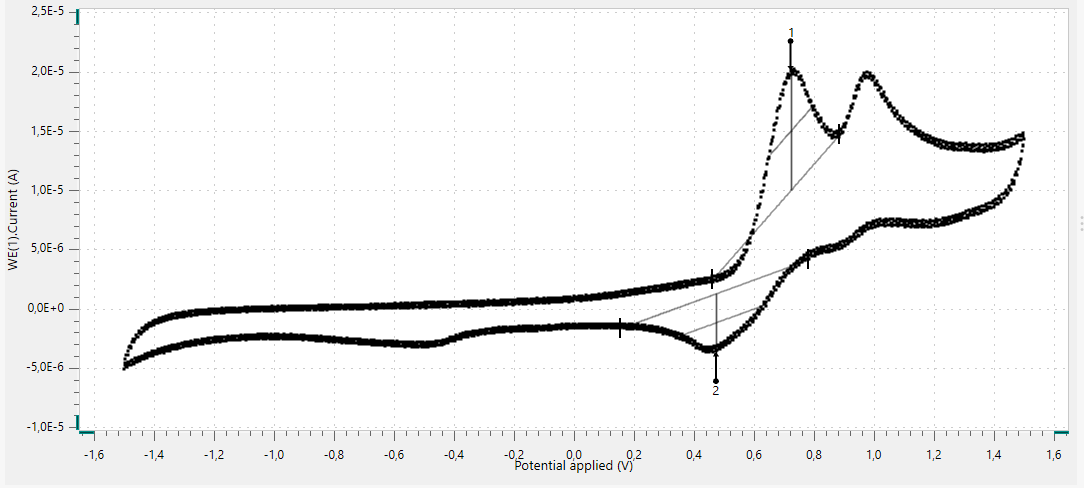
**

**Supplementary Figure 8:** Cyclic voltammogram of propyl gallate in methanol.

**Supplementary Figure 9:** Chromatogram obtained with a 1000 µg/mL EXE aqueous solution using Assay RS method


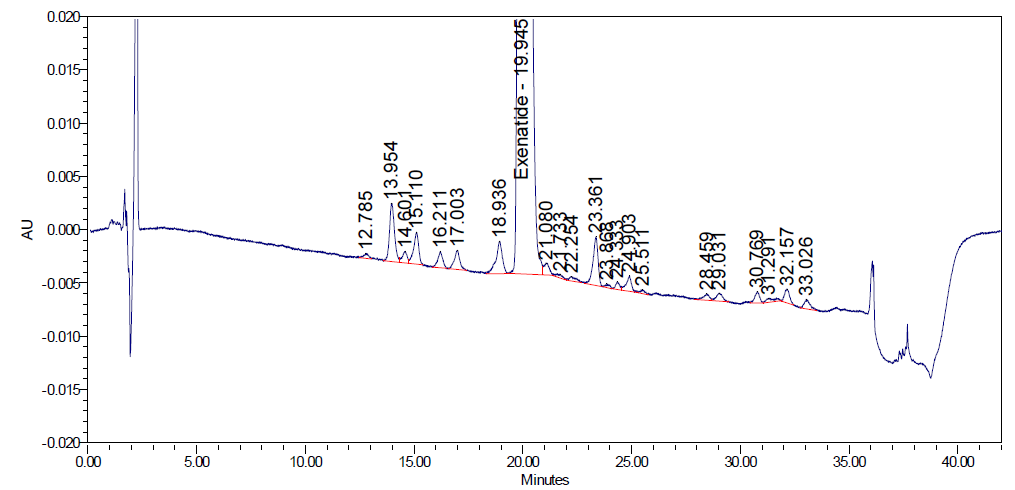


**Supplementary Table 2: Relative retention time of Exenatide impurities listed in USP monograph**

| **Name** | **Relative retention time listed in Exenatide USP monograph** | **Relative retention time with developed Assay RS method** |
| --- | --- | --- |
| [Glu13]-exenatide | 0.65–0.68 | 0.64 |
| Sum of [Asp28]-exenatide and  [Met(O)14]-exenatide | 0.68–0.76 | 0.70-0.76 |
| Exenatide | 1.00 | 1.00 |

**Supplementary Table 3: Percentage area of chromatogram peaks obtained with Assay RS method during stability study, expressed as mean ± SD (n=3)**

|  | **Time / condition** | **[Glu^13^]-EXE (%)** | **[Asp^28^]-EXE+ [Met(O)^14^]-EXE (%)** | **EXE (%)** | **Others impurities (%)** | **Total Impurities (%)** |
| --- | --- | --- | --- | --- | --- | --- |
| EXE | T0 | 1.08 ± 0.01 | 0.89 ± 0.01 | 92.24 ± 0.13 | 5.79 ± 0.13 | 7.76 ± 0.13 |
| HIP | T0 | 1.15 ± 0.03 | 1.08 ± 0.08 | 91.34 ± 0.34 | 6.43 ± 0.23 | 8.66 ± 0.31 |
| HIP F9 | T0 | 0.08 ± 0.02 | 2.48 ± 0.06 | 92.39 ± 0.27 | 5.05 ± 0.25 | 7.62 ± 0.27 |
|  | T4W 5°C | 0.25 ± 0.01 | 3.92 ± 0.11 | 89.64 ± 0.63 | 6.19 ± 0.62 | 10.36 ± 0.63 |
|  | T4W RT | 0.16 ± 0.03 | 5.86 ± 0.04 | 81.36 ± 0.26 | 12.62 ± 0.30 | 18.64 ± 0.26 |
| HIP F9 C10 | T0 | 0.14 ± 0.04 | 2.37 ± 0.15 | 88.48 ± 0.80 | 9.02 ± 0.92 | 11.52 ± 0.80 |
|  | T4W 5°C | 0.61 ± 0.04 | 3.25 ± 0.02 | 83.66 ± 0.21 | 12.48 ± 0.21 | 16.34 ± 0.21 |
|  | T4W RT | 0.56 ± 0.03 | 6.35 ± 0.12 | 75.08 ± 0.35 | 18.01 ± 0.23 | 24.92 ± 0.35 |
| EXE.Ac F9 | T0 | 0.18 ± 0.01 | 1.78 ± 0.03 | 95.99 ± 0.09 | 2.05 ± 0.10 | 4.01 ± 0.09 |
|  | T4W 5°C | 0.12 ± 0.01 | 2.27 ± 0.06 | 92.83 ± 0.31 | 4.78 ± 0.31 | 7.17 ± 0.31 |
|  | T4W RT | 0.17 ± 0.0è | 3.44 ± 0.13 | 92.35 ± 0.47 | 4.03 ± 0.29 | 7.65 ± 0.47 |
| EXE.Ac F9 C10 | T0 | 0.07 ± 0.01 | 1.85 ± 0.07 | 93.92 ± 0.14 | 4.15 ± 0.13 | 6.20 ± 1.39 |
|  | T4W 5°C | 0.14 ± 0.01 | 2.34 ± 0.04 | 94.41 ± 0.34 | 3.11 ± 0.28 | 5.59 ± 0.25 |
|  | T4W RT | 0.61 ± 0.06 | 3.39 ± 0.09 | 85.09 ± 0.18 | 10.91 ± 0.16 | 14.91 ± 0.18 |
